# Piglet nasal microbiota at weaning may influence the development of Glässer’s disease during the rearing period

**DOI:** 10.1186/s12864-016-2700-8

**Published:** 2016-05-26

**Authors:** Florencia Correa-Fiz, Lorenzo Fraile, Virginia Aragon

**Affiliations:** IRTA, Centre de Recerca en Sanitat Animal (CReSA, IRTA-UAB), Campus de la Universitat Autònoma de Barcelona, 08193 Bellaterra, Spain; Departament de Producció Animal, ETSEA, Universitat de Lleida, 25198 Lleida, Spain

**Keywords:** Nasal microbiota, Swine microbiota, Glässer’s disease, Bacterial diversity

## Abstract

**Background:**

The microbiota, the ensemble of microorganisms on a particular body site, has been extensively studied during the last few years, and demonstrated to influence the development of many diseases. However, these studies focused mainly on the human digestive system, while the populations in the respiratory tract have been poorly assessed, especially in pigs. The nasal mucosa of piglets is colonized by an array of bacteria, many of which are unknown. Among the early colonizers, *Haemophilus parasuis* also has clinical importance, since it is also the etiological agent of Glässer’s disease. This disease produces economical losses in all the countries with pig production, and the factors influencing its development are not totally understood. Hence, the purpose of this work was to characterize the nasal microbiota composition of piglets, and its possible role in Glässer’s disease development.

**Results:**

Seven farms from Spain (4 with Glässer’s disease and 3 control farms without any respiratory disease) and three farms from UK (all control farms) were studied. Ten piglets from each farm were sampled at 3–4 weeks of age before weaning. The total DNA extracted from nasal swabs was used to amplify the 16S RNA gene for sequencing in Illumina MiSeq. Sequencing data was quality filtered and analyzed using QIIME software. The diversity of the nasal microbiota was low in comparison with other body sites, showing a maximum number of operational taxonomic units (OTUs) per pig of 1,603, clustered in five phyla. Significant differences were found at various taxonomical levels, when the microbiota was compared regarding the farm health status. Healthy status was associated to higher species richness and diversity, and UK farms demonstrated the highest diversity.

**Conclusions:**

The composition of the nasal microbiota of healthy piglets was uncovered and different phylotypes were shown to be significantly altered in animals depending on the clinical status of the farm of origin. Several OTUs at genus level were identified over-represented in piglets from control farms, indicating their potential as probiotics. Although we provide relevant data, fully metagenomic approaches could give light on the genes and metabolic pathways involved in the roles of the nasal microbiota to prevent respiratory diseases.

**Electronic supplementary material:**

The online version of this article (doi:10.1186/s12864-016-2700-8) contains supplementary material, which is available to authorized users.

## Background

Many body sites from mammalian species provide habitats for a huge number of microbial species. Studies on microbiota, the microbial composition of a body site, have increased over the last years with the arrival of NGS technology. The influence of the microbiota composition on the development of many diseases [1–3], together with the possibility of manipulating that composition to control disease [4–7]), have conferred a central role to these studies during the last few years. Most of the microbiota studies have been done in humans, although other species are also being investigated [8–11]. Additionally, the majority of the studies have focused on the bacterial gut populations, through the analysis of intestinal tract or feces, although body sites such as skin, mouth, nose or vagina have also been investigated [12–15]. Other sites related to respiratory diseases have been also analyzed, such as lungs or tonsils; especially in animal models due to invasive nature of the sampling process [16–18].

Among the animal models, pigs are important for society, mainly because pork is the world’s most consumed meat from terrestrial animals (http://www.fao.org). Pig industry has risen globally and the emergence of specific zoonotic diseases is of increasing concern. Pigs can act as reservoirs of several pathogens becoming a risk to farmers and consumers. Moreover, because pigs have demonstrated to be one of the best models for human diseases [19, 20], the unraveling of the microbiota composition is of great interest.

Most microbiota studies have focused on species related to digestive system while the populations related to respiratory have been poorly assessed for pigs. The tonsils have historically been used to explore the upper respiratory microbial populations, but the fact that nose is the first place where the microbes find a barrier to infection, studies on nasal microbiota can be relevant. The nose can harbor important microorganisms that can be pathogenic under certain circumstances.

Among the microbes widely distributed in swine population, *Haemophilus parasuis* is a small, pleomorphic, Gram-negative rod that colonizes the upper respiratory tract of healthy piglets [21]. However, under some conditions (stress, lack of immunity, presence of other pathogens,...), virulent strains can spread to the lung and invade systemic sites, causing Glässer’s disease [22, 23], which is characterized by fibrinous polyserositis, including meningitis. Glässer’s disease is considered one of the bacterial diseases with highest economic impact in nursery piglets (4–8 weeks of age) and it is present in all the major swine-raising countries. This disease is normally controlled by antimicrobial treatments; however antibiotic therapies have been shown to select resistant *H. parasuis* strains in farms [21]. Moreover, antimicrobial treatments can deplete members of the resident commensal bacterial population, which may be beneficial in preventing colonization with virulent *H. parasuis*. Actually, many members of the microbiota are known to be selected by the host to prevent colonization by pathogens, and that could be the case for *H. parasuis*. The knowledge of the composition of the microbiota of animals from farms without respiratory diseases and the potential differences with animals from farms with Glässer’s disease outbreaks will be of enormous relevance to better understand this disease and its control. Moreover, the different microorganisms from healthy animals can be used to further study their potential benefits.

Here, we report the nasal microbiota composition of pigs at weaning and its potential relation to susceptibility to respiratory conditions, such as Glässer’s disease.

## Results

### The nasal microbiota of piglets from control and Glässer’s diseased-farms

Samples subjected to individual massive sequencing were taken from ten pigs per farm at 3–4 weeks of age, just before weaning. We analyzed samples from animals from four farms with Glässer’s disease outbreaks (GD farms) and from six control farms without any respiratory condition reported (C farms). *H. parasuis* colonize piglets at the early beginning of life, but Glässer’s disease normally develops at 4–8 weeks of age, after weaning. Thus, the analysis was performed with samples taken at 3–4 weeks of age to study the possible predisposition to disease.

We obtained a total of 37,376,035 sequences from four different runs. The reads were joined and the erroneous or poor quality reads (Q < 25) were eliminated. This left us with 45 ± 18 % of the reads, corresponding to a median number of reads per sample of 232,807 (range 7,338–844,521) with a mean sequence length of 456.77 ± 18.6 nt. Singletons and samples with low coverage (less than 40,000 reads per sample) were discarded, so as to have a more representative and homogenous data.

**Fig. 1:**
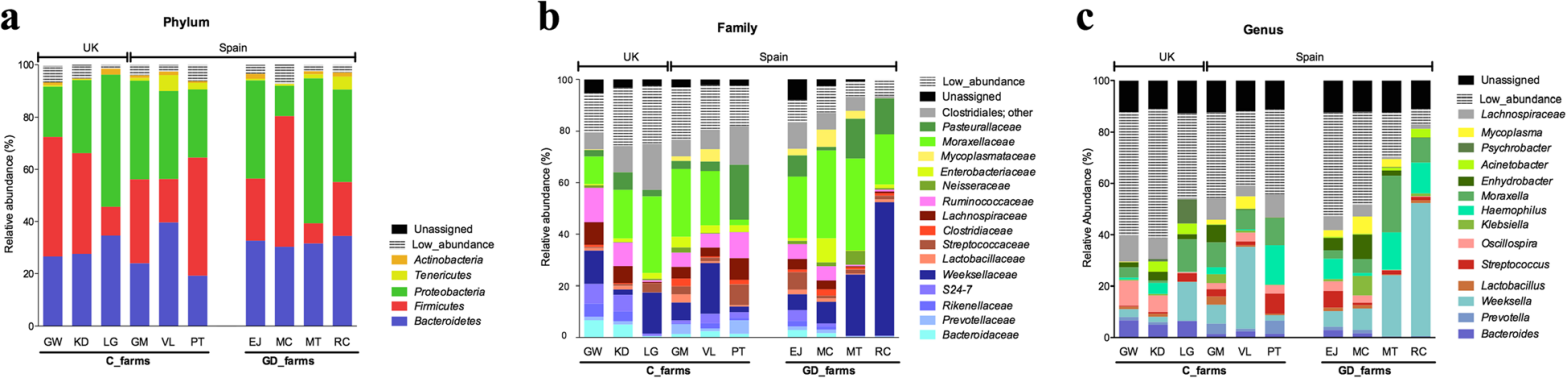
Nasal microbiota of piglets from farms with Glässer’s disease (GD) and control farms (C). The mean relative abundance (%) of OTUs found in nasal swabs of 3–4 weeks-old piglets is presented. Samples were analyzed altogether (Total), or were grouped based on the health status (C and GD). Each graph represents the OTUs at different taxonomical levels: phylum (**a**), family (**b**), genus (**c**). Results from individual farms are presented in Additional file 1

OTUs were identified in all samples by clustering sequences at 97 % sequence homology. The maximum number of OTUs clustered per pig was 1603 OTUs, where 92.1 % of the OTUs were classified in only 3 phyla, leaving 2.3 % of sequences without classification at phylum level. The relative abundance of the OTUs found in the nasal microbiota is represented in Fig. 1 (see also Additional file 1). The diversity of the nasal microbiota was as low as was reported before in different animal and in human studies [24–27]. Within the phyla found in nasal cavities of pigs, globally, *Proteobacteria* represented a mean of 37.0 %, *Bacteroidetes* 28.6 % and *Firmicutes* 26.5 % (Fig. 1, ‘total’). Other two phyla with more than 1 % of relative abundance were *Tenericutes* with 2.2 % and *Actinobacteria* with 1.3 %. Within *Proteobacteria*, the *Moraxellaceae* family was the most abundant, representing 22.8 % of all the families found (42.4 % of the assigned as *Proteobacteria* phylum). *Pasteuracellaceae* was also abundant within the *Proteobacteria* phylum, with 6.9 % of relative abundance. Globally, the second most abundant family found was *Weeksellaceae* with 15.3 %, which represented 63.7 % of the sequences in the *Bacteroidetes* phylum. Interestingly, *Firmicutes* was more homogeneously divided in different families, with *Ruminicoccaceae* being the most abundant (mean relative abundance 6.1 %) followed by *Lactobacillaceae* (4.3 %) and *Streptococcaceae* (2.5 %). The family *Mycoplasmacetae* represented 90 % of the *Tenericutes* phylum. The percentage of unassigned sequences (or assigned as ‘other’) at family level was 12.6 %.

The most prevalent genus found in the nasal microbiota was *Moraxella* (17.2 %), followed by *Weeksella* (12.9 %). *Haemophilus* was also abundant, representing 6.1 % of the reads classified at genus level, and 27.0 % of the *Pasteurallaceae* family. Almost half of the genera in the *Lactobacillaceae* family were *Lactobacillus*, with 49.0 % of relative abundance within this family. Unfortunately, not all sequences could be confidently assigned to one genus (21.7 % assigned as ‘other’) and may deserve further studies to be characterized.

To unravel the differences in nasal microbiota in piglets from farms with a good healthy status (C farms) in contrast to those from farms with Glässer’s disease (GD farms), we analyzed the relative abundance of OTUs at three main levels (phylum, family and genus) by grouping samples according to the health status of the farm of origin (Fig. 1). Figure 1 shows the averaged relative abundance per health status in two groups: GD and C farms. At phylum level, there is a relative increase in *Proteobacteria* (32.5 % in C to 44.0 % in GD) in conjunction to a decrease in *Firmicutes* (21.1 to 18.5 %) in animals from farms with Glässer’s disease. The increase in *Proteobacteria* corresponds to a higher abundance of *Pasteurellaceae* (5.1 % vs 9.5 % in C and GD, respectively) and *Moraxellacea* (19.3 % vs 27.8 % in C and GD, respectively) families. Within the *Pasteurellaceae* family, the genus *Haemophilus* was found increased in pigs from farms with Glässer’s disease (2.8 % vs 9.4 % in C and GD, respectively). Here, *Haemophilus* corresponds to the unique porcine species of this genus, *Haemophilus parasuis,* the etiological agent of Glässer’s disease. The *Moraxellaceae* family showed a raised amount of the genera *Moraxella* (13.6 to 22.2 % in C and GD, respectively) and *Enhydrobacter* (3.1 to 4.3 % in C and GD, respectively). Other bacterial components of the microbiota, although present in low abundance, deserve further investigation due to their absence in animals from diseased farms. This is the case for *Psychrobacter*, from the *Moraxellaceae* family, which was only present in piglets from C farms (at 1.8 %). In addition, within *Firmicutes*, the principal changes were seen in the *Ruminococcaceae* family, which decreased from 8.0 % in C farms to 3.4 % in GD ones. These reductions are the reflection, at least in part, of the diminished amount of *Oscillospira* (4.5 to 1.8 % in C and GD, respectively) and *Ruminococcus*, with 1.0 % in animals from C farms but absent in GD farms. *Lachnospiraceae* family showed a significant decrease in GD farms (2.9 to 1.1 %), which was due to many low-abundant genera that diminished simultaneously. Many of the changes observed in *Firmicutes* could not be associated to any family, because the sequence assignment was done confidently only until order level, and corresponded to *Clostridiales*. In contrast to this general decreased behavior in *Firmicutes*, the *Streptococcaceae* family showed a significant raise, revealed by the increased tendency observed in *Streptococcus* (from 2.1 to 2.9 % in C and GD, respectively).

**Fig. 2:**
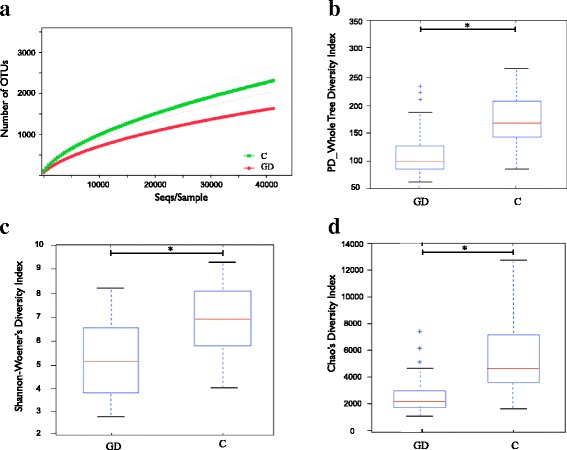
Alpha diversity on rarefied samples analyzed by health status. Species richness of nasal samples from farms with Glässer’s disease (GD) and control farms with no respiratory condition (C); dotted lines represent the standard deviation (**a**). Alpha diversity was compared between groups by measuring different metrics: PD_Whole Tree (**b**), Shannon-Wiener’s (**c**), and Chao1 (**d**). Outliers are indicated with plus signs on the plot. * denotes *P* ≤ 0.05. Results from individual farms are presented in Additional file 2

To measure richness within each farm and/or within healthy status condition, rarefactions were performed considering the maximum depth. At this maximum subsampling depth, the mean observed species richness was 2,548 and 1,689 species for C and GD farms, respectively (Fig. 2). In all samples the plateau was reached at the maximum depth, meaning that the sample procedure was adequate. Samples corresponding to pigs from C farms showed higher species richness, as it is depicted in Fig. 2. Analysis of alpha diversity metrics, the Shannon’s and Chao confirmed the lower diversity in the nasal microbiota of piglets from diseased farms, which demonstrated to be statistically significant through non-parametric *t*-test (999 permutations, *p* < 0.05, Fig. 2). The Faith’s PD Whole Tree metrics, which includes phylogenetic information about the evolutionary distance between taxa (since it accounts for the structure of the phylogenetic tree), was also measured and demonstrated to be in agreement to the other metrics (Fig. 2). Farms from UK showed the higher diversity in all cases. The alpha diversity from different farms was also investigated (Additional file 2) and showed an association of higher richness on rarefied samples from UK farms (*t*-test with Monte Carlo permutation, *p* < 0.05). Farm GW (C, from UK) showed the highest number of observed species and farm RC (GD, from Spain) the lowest.

### Effect of health status, country and farming company on beta diversity

To understand the differences across the microbiota on nasal samples, the beta diversity was estimated. Weighted and unweighted UniFrac phylogenetic distances were used to generate the beta diversity distance matrices and calculate the degree of differentiation among the samples. Samples were grouped according to different characteristics to test them as possible factors leading to clustering.

**Fig. 3:**
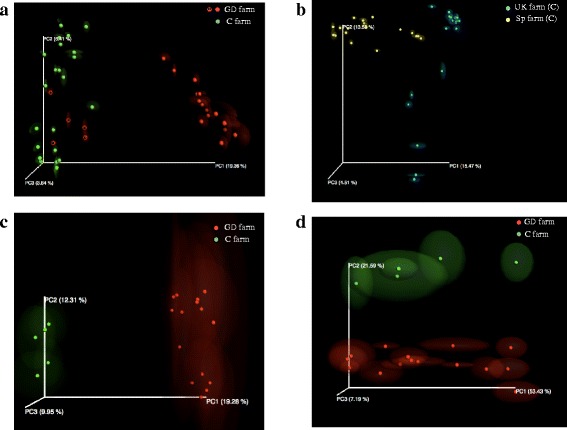
Principal Component Plots (jackknifed) representing beta diversity on rarefied samples. Beta diversity of nasal samples of piglets was computed through unweighted UniFrac analysis for each of the following groups: animals from Spanish farms with different clinical status (**a**), animals from control farms without respiratory diseases from Spain and UK (**b**), animals sharing sow origin but with different health status (**c**). The weighted UniFrac analysis of samples from animals sharing sow origin but with different health status is also shown (**d**). In (**a**), red spheres with a white dot correspond to animals that remained healthy throughout the study from a farm (EJ) with Glässer’s disease (GD)

In order to study the effect of farm health status only Spanish farms were included in the beta diversity analysis. The effect of country of origin was analyzed separately, comparing only farms from Spain or the UK with no respiratory condition reported; in this case avoiding the health-status factor. In addition, the microbiota composition has been positively correlated with the genetic background in different hosts [28–32]. Although establishing the specific genetic background was out of the scope of this work, we recorded the farming company that distributes the sows (sow origin) at the sampling farms. Three farms, two C and one GD farms have the same sow origin, so we analyzed this group separately, too. Principal coordinate analysis was done on each group and resampling was performed repeatedly on a subset of the available data of each sample evenly (jackknifing) to measure the robustness of individual clusters in PCoA plots. The emperor PCoA plots obtained are depicted in Fig. 3. To test for the percentage of variation of samples in each group, *R*^2^ was measured using Adonis from Vegan package, a nonparametric statistical method [33–35]. Regarding the health status in Spanish farms, the mean distances between two groups (C and GD Spanish farms) were calculated and showed to be statistically different in the unweighted analysis (Fig. 3a; *R*^2^ = 0.09787, *p* = 0.01), showing a qualitatively different composition of the microbiota according to the health status of the farm. When the country of farm location was evaluated, differences in clustering between these two groups were also statistically significant (Fig. 3b; *R*^2^ = 0.1038 *p* = 0.01). However, farms from UK and Spain can be under different management conditions, such as different antimicrobial treatment, so the differences observed between the two countries cannot be directly associated to localization and further studies should be done.

When we focus on the 3 farms with the same sow origin, the qualitative differences observed between farm health status was significant, with *R*^2^ = 0.1821 (Fig. 3c), explaining 18 % of the variation between groups, the highest observed in all cases. Based on these results, the largest source of the variation in bacterial community composition between samples may be attributed to differences in sow origin, which could be associated to genetic background.

The weighted analysis did not show differences when comparing country of origin or health status, indicating that the differences observed between groups (those demonstrated through the unweighted analysis) may correspond to species in lower abundance, as the weighted UniFrac emphasizes common OTUs and deemphasizes rare OTUs [36, 37]. However, when analyzing only samples from the same sow origin separately, both weighted (Fig. 3d) and unweighted (Fig. 3c) UniFrac distances showed good clustering according to health condition (weighted Unifrac analysis, *R*^2^ = 0.1917 *p* < 0.05; unweighted UniFrac analysis, *R*^2^ = 0.2222 *p* < 0.05). Thus, sow origin may be a relevant factor to determine the nasal microbiota composition in pigs, whose modifications would affect the health status of the piglets.

### The core microbiota in Spanish and British farms

Differences at several taxonomy levels were found in the nasal microbiota composition of piglets depending on the country of origin. However, some of these were due to low abundant OTUs, which may be a result of the specific moment of sampling rather than colonization of the particular taxon. The analysis of the core microbiome, defined as those OTUs present in all the samples from C farms, was made with the aim of finding permanent/essential inhabitants of the nasal cavity of pigs (colonizers).

**Fig. 4:**
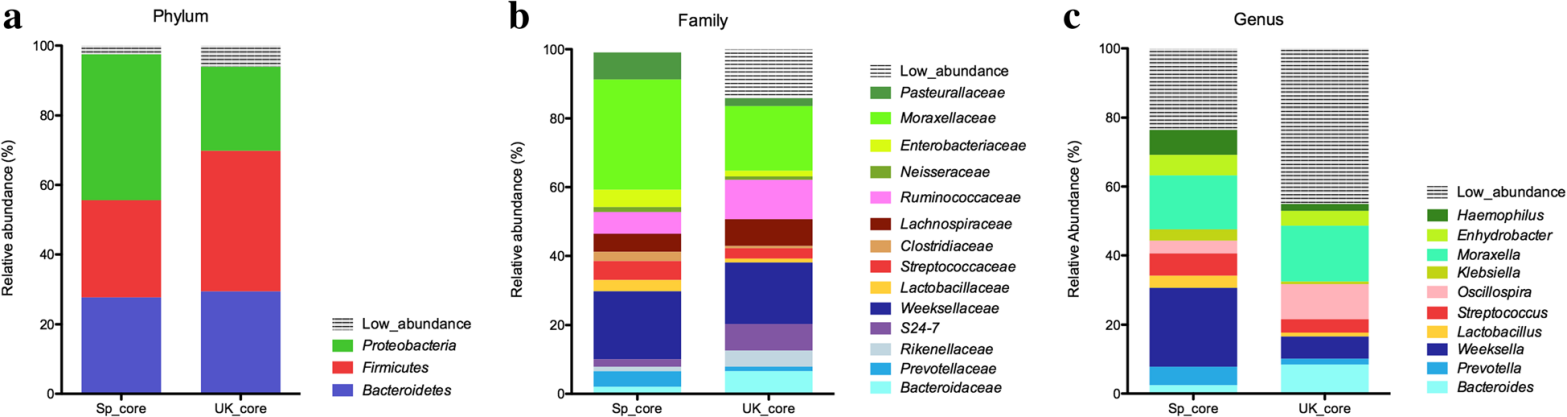
The core of the nasal microbiota of piglets from Spanish (Sp) and British (UK) farms. Bars represent relative abundance for the OTUs found in all healthy animals at a minimum of 1 %. Each graph represents the OTUs at different taxonomical levels: phylum (**a**), family (**b**), genus (**c**). Unassigned sequences were not included in the core analysis

In order to study the effect of localization (country) on the nasal microbiota, the nasal core microbiota was determined for both Spanish and British farms separately (OTUs with >1 % relative abundance present in all C pigs). In agreement with the alpha diversity analysis, the UK core showed higher number of identified OTUs than the Spanish core (54 in Spain *vs* 72 in UK farms). The relative abundance observed for three different taxonomic levels (phylum, family and genus) for both the British (UK) and the Spanish (Sp) cores are shown in Fig. 4. The common OTUs from the core that were differentially present in the samples from the two countries are shown in Table 1. There were remarkably differences in the core OTUs found in control Spanish farms in comparison to UK farms. At phylum level, in the Sp core *Bacteroidetes* (mean = 27.6 %) and *Firmicutes* (mean = 28.0 %) were present at similar levels, while *Proteobacteria* was the most abundant phylum, with a mean of 41.8 % of the total reads classified at this level. In the UK core, however, the most abundant phylum was *Firmicutes* with 40.5 %, while *Bacteroidetes* (mean = 29.3 %) and *Proteobacteria* (mean = 24.1 %) were detected in similar abundance. The main differences within *Proteobacteria* were associated to two families: *Pasteuralleaceae* and *Moraxellaceae,* which showed to be in lower abundance in the UK core (Fig. 4, Table 1). At genus level, the difference within the *Pasteuralleaceae* family was directly related to a decrease of *Haemophilus* in the UK core (Sp core mean = 9.2 % vs UK core mean = 2.0 %). Unfortunately the difference within the *Moraxellaceae* family (Sp core mean = 31.9 %, UK core mean = 18.7 %) could not be attributed to any particular genus, due to the lack of identification of the differential OTUs at this level. The OTUs that were identified at genus level within *Moraxellaceae* (*Enhydrobacter* and *Moraxella*) were not accountable for the differences observed in the family, as they were represented in similar abundance in the two cores (Sp core vs UK core; *Enhydrobacter*: 7.2 % vs 6.2 %; *Moraxella*: 19.0 % vs 19.6 %).

**Table 1:** Relative abundance of significantly different OTUs of the healthy core from Spanish (Sp) and British (UK) farms

Taxonomy					Relative abundance (%)	
*Phylum*	*Class*	*Order*	*Family*	*Genus*	Sp core	UK core
*Bacteroidetes*					27.6202	29.3460
	*Bacteroidia*	*Bacteroidales*			12.3122	23.0031
			*Rikenellaceae*		1.3096	4.6966
			*S24-7*		2.1583	7.7633
			*Prevotellaceae*		5.5314	1.7105
				*Prevotella*	5.3730	1.6850
			*Odoribacteraceae*		0.1273	1.0529
				*Odoribacter*	0.1273	1.0529
			*Bacteroidaceae*		2.5661	8.5857
				*Bacteroides*	2.4499	8.4674
	*Flavobacteriia*	*Flavobacteriales*	*Weeksellaceae*		19.6512	17.7220
				*Weeksella*	21.7857	6.4157
*Deferribacteres*					0.3435	1.3969
	*Deferribacteres*	*Deferribacterales*	*Deferribacteraceae*		0.3435	1.3969
				*Mucispirillum*	0.3435	1.3969
*Cyanobacteria*					0.4103	1.1756
	*4C0d-2*	*YS2*			0.2043	1.1571
*Firmicutes*					28.0177	40.4671
	*Clostridia*	*Clostridiales*			4.4292	12.2484
			*Clostridiaceae*		2.7209	0.6808
			*Lachnospiraceae*		5.2361	7.7619
			*Ruminococcaceae*		6.3337	11.4209
				*Oscillospira*	3.6856	10.0053
				*Ruminococcus*	0.6039	1.3945
	*Bacilli*	*Lactobacillales*	*Lactobacillaceae*		3.3117	1.1864
				*Lactobacillus*	3.3056	1.1826
			*Streptococcaceae*		5.5737	3.9812
				*Streptococcus*	5.4903	3.8330
			*Aerococcaceae*		0.2347	1.2245
				*Aerococcus*	0.2036	1.1799
*Proteobacteria*					41.8425	24.0760
	*Alphaproteobacteria*	*RF32*			0.3869	1.8664
	*Betaproteobacteria*	*Neisseriales*	*Neisseriaceae*		1.4205	1.0527
	*Gammaproteobacteria*	*Enterobacteriales*	*Enterobacteriaceae*		5.0675	1.6001
		*Pasteurellales*	*Pasteurellaceae*		7.8926	2.3031
				*Haemophilus*	9.2309	1.9811
		*Pseudomonadales*	*Moraxellaceae*		31.9200	18.7081

The increased number of members within *Firmicutes* in the UK core was demonstrated to be related mainly to the order *Clostridiales* (Table 1). Although many of these differences can be associated to several genera, more studies should be made to make a step ahead to understand the species involved.

The apparent lack of changes in *Bacteroidetes* between the UK and Sp cores are the result of several compensatory modifications at family or genus levels. *Bacteroidales* order was increased in the UK core, with an increment in the *Bacteroidaceae* family and *Bacteroides* genus. Also, *Rikenallaceae* and S24-7 families showed increased levels in UK core in contrast to what is observed in Sp core. Concomitantly, an opposite tendency was observed for the *Flavobacteriales* order, which was increased in the Sp core, due mainly to an increase in the *Weeksellaceae* family, mostly related to an increase in *Weeksella*.

The genus *Myroides* (from the *Flavobacteriaceae* family) was especially abundant in most UK farms while absent in Spain, probably being specifically related to location (data not shown). However, this result is not found in the core analysis, because *Myroides* was absent in some animals from a particular farm. These particularly abundant taxa deserve further investigation on the potentially protective role they may have against diseases.

### Comparison of relative abundance of core nasal microbiota composition of animals from farms with different health status

With the aim of finding microbes that could be associated to health on piglets, the core nasal microbiota composition of C pigs was compared with GD pigs, only in farms from Spain. The presence of the particular OTUs found was also contrasted in UK farms. Moreover, we decided to analyze the nasal microbiota composition of wild boars (*Sus scrofa*) from Catalonia (Spain). There is little information available regarding *H. parasuis* infection in these animals with only one report showing evidence on Glässer’s disease [38]. So, the presence of a particular OTU in nasal microbiota of healthy pigs (*Sus scrofa* domesticus), absent in those pigs from farms with Glässer’s outbreaks, would become more relevant if found also in UK farms and/or in wild boars, as they seem to be more resistant to the disease [39].

Table 2 shows the relative abundance of only those OTUs from C farms that were found to be statistically different when compared to GD farms from Spain (g-test, *p* < 0.05). Also the relative abundance in UK farms and in wild boars was included for comparison. Notably, *Bacteroides*, which was shown to be enriched in farms from UK, appeared increased in animals from C farms when compared to GD farms, and presented a relative abundance of 2.4 % in healthy wild boars. Other genera that became relevant because of their increased proportion in healthy animals, such as *Oscillobacter* and *Lactobacillus* (both from *Clostridiales* order) were also present in wild boars and UK farms, although in low abundance. *Prevotella* appeared as an important member of pigs form C farms, which was also present in high abundance in wild boars. With an opposite behavior, *Streptococcus* showed an increased tendency in GD farms with low abundance in UK farms or wild boars. *Haemophilus* was found in high abundance in wild boars and GD farms, with significantly lower levels in control farms from both UK and Spain.

**Table 2:** Differential OTUs in diseased (GD) and control (C) Spanish farms; comparison with wild boars and UK farms

Taxonomy					Relative abundance (%)			
					C (Spain)	GD (Spain)	H (UK)	Wild Boars (Spain)
*Phylum*	*Class*	*Order*	*Family*	*Genus*	%	%	%	%
*Bacteroidetes*					26.1250	29.1804	29.346	23.8059
		*Bacteroidales*	*Bacteroidaceae*	*Bacteroides*	1.6932	1.3426	8.4674	2.385
			*Porphyromonadaceae*		2.8115	2.2021	0.6906	0.5237
				*Barnesiella*	1.8488	1.4950	<0.1	<0.1
			*Prevotellaceae*		4.3225	1.3013	1.7105	2.5665
				*Prevotella*	3.3216	0.9066	1.685	2.5118
			*Weeksellaceae*		14.9059	21.7160	17.7222	1.5944
				*Weeksella*	14.5530	20.2616	6.4157	<0.1
*Firmicutes*					30.3821	19.0583	40.4671	20.4018
	*Bacilli*	*Lactobacillales*	*Lactobacillaceae*		1.9351	1.2777	1.1864	0.4151
				*Lactobacillus*	1.9305	1.1653	1.1826	0.4134
			*Streptococcaceae*		2.9158	3.6426	3.9812	0.2754
				*Streptococcus*	2.8840	3.6244	2.833	0.2714
	*Clostridia*	*Clostridiales*			12.3320	7.6941	12.2484	18.8775
			*Lachnospiraceae*		8.7595	5.4816	7.7619	4.2165
				*Clostridium XlVa*	2.5326	1.6417	<0.1	<0.1
			*Peptostreptococcaceae*		1.2544	0.7615	<0.1	<0.1
				*Clostridium XI*	1.1982	0.7349	<0.1	<0.1
			*Ruminococcaceae*		7.8575	3.5140	11.4209	5.3328
				*Oscillibacter*	1.5302	0.9355	<0.1	<0.1
*Proteobacteria*					36.9336	44.1926	24.076	39.6401
	*Betaproteobacteria*	*Neisseriales*	*Neisseriaceae*		1.1309	2.0309	1.0527	0.2597
				*Neisseria*	0.7160	1.3641	<0.1	<0.1
	*Gammaproteobacteria*	*Enterobacteriales*	*Enterobacteriaceae*		3.6917	3.0032	1.6001	0.8243
				*Klebsiella*	2.1640	2.7505	<0.1	0.3157
		*Pasteurellales*	*Pasteurellaceae*		6.4292	9.5108	2.3031	11.5475
				*Actinobacillus*	1.4163	1.4664	<0.1	0.2603
				*Haemophilus*	3.6043	7.6630	1.9811	11.1102
				*Pasteurella*	1.3354	0.0468	<0.1	<0.1
		*Pseudomonadales*	*Moraxellaceae*		23.5127	27.8645	18.7081	20.4429
				*Acinetobacter*	0.1482	1.2382	1.8639	5.7347
				*Moraxella*	23.2688	26.4523	16.2312	12.285
*Tenericutes*					2.3707	3.1578	0.4713	1.9082
	*Mollicutes*	*Mycoplasmatales*	*Mycoplasmataceae*		2.2363	3.0477	0.1365	1.5609
				*Mycoplasma*	2.2361	3.0476	0.1356	1.5609

As stated before, Glässer’s disease usually develops after weaning, around 4–8 weeks of age. The samples sequenced in this work were taken at 3–4 weeks of age to test for the predisposition of getting the disease. The clinical symptoms were followed in piglets from farm EJ (Spain) for 3 months. From the ten animals sampled, five showed clinical symptoms of Glässer’s disease at 8 weeks after weaning, while the other five did not. The bacterial communities present at these animals that remained healthy from Glässer’s disease, grouped closely to the samples from animals from control farms denoting to have more similarity with them. The samples from EJ farm that remained healthy are indicated with a white dot inside the red spheres in Fig. 3a. This result denotes that the nasal microbiota composition of piglets at 3–4 weeks of age differed in piglets within the same farm. The OTUs that significantly differed between these two groups are shown in Table 3. The most abundant OTU at family level was *Moraxellaceae*, which was higher in animals that developed respiratory symptoms, and the main difference was determined by the *Moraxella* genus (29.5 % *vs* 17.1 %; *p* < 0.05). The nasal microbiota was enhanced in *Pasteurallaceae* in those animals that got sick, in particular in *Haemophilus* (2.5 % *vs* 11.4 %; *p* < 0.05). This increased composition was observed in conjunction with increments in other common opportunistic pathogens such as *Staphylococcus* (0.3 to 1.4 %) and *Streptococcus* (3.5 to 9.5 %). Contrary to this tendency, *Bacteroidaceae* family showed a significant decrease in animals that later developed disease, with *Bacteroides* being the genera responsible of this observation within this taxonomy level (4.6 to 1.2 %). Other genera showed a decrease tendency and deserve further characterization as they could become key players in protection against this disease (*Barnesiella*, *Prevotella*, *Alistipes*, *Oscillobacter*, *Pseudoflavonifractor,* among others). Of special interest is the *Clostridiales* order, with several families showing decreased tendencies in those animals that remained healthy (i.e. *Lachnospiraceae* and *Ruminococcus*). Several *Clostridium* genera showed a decreased abundance associated to health. Other pathogen that showed to be increased in animals that developed clinical symptoms of Glässer’s disease was *Mycoplasma,* which showed the same behavior when C and GD farms were compared globally (Table 2).

**Table 3:** Differential OTUs in healthy or diseased animals from a Spanish farm (EJ) with Glässer’s disease (GD)

Taxonomy					Reative abundance (%)	
					EJ (Spain)	EJ (Spain)
*Phylum*	*Class*	*Order*	*Family*	*Genus*	Glässer developed	Remained healthy
*Actinobacteria*					3.5894	2.1710
	*Actinobacteria*	*Actinomycetales*	*Corynebacteriaceae*		1.9080	0.7514
				*Corynebacteria*	1.9067	0.7491
*Bacteroidetes*					13.5458	26.7920
	*Bacteroidia*	*Bacteroidales*	*Bacteroidaceae*		1.2506	4.6349
				*Bacteroides*	1.2499	4.6337
			*Porphyromonadaceae*		2.3659	8.3669
				*Barnesiella*	1.6559	6.0845
			*Prevotellaceae*		1.5082	2.6173
				*Prevotella*	1.0668	1.4726
			*Flavobacteriaceae*		6.8750	5.6079
				*Planobacterium*	2.7625	1.5614
			*Weeksellaceae*		4.1125	4.0465
				*Weeksella*	3.3241	3.4334
			*Rikenellaceae*		0.8218	3.5809
				*Alistipes*	0.8218	3.5809
*Firmicutes*					27.6055	40.7701
	*Bacilli*	*Bacillales*	*Staphylococcaceae*		1.4925	0.3198
				*Staphylococcus*	1.4091	0.2708
		*Lactobacillales*	*Lactobacillaceae*		2.0031	1.9052
				*Lactobacillus*	1.6017	1.6558
			*Streptococcaceae*		9.6119	3.5364
				*Streptococcus*	9.5011	3.4847
			*Leuconostocaceae*		1.2007	0.4920
	*Clostridia*	*Clostridiales*			11.4187	32.7266
			*Lachnospiraceae*		4.8052	18.2475
				*Clostridium XlVa*	1.4577	5.2715
			*Ruminococcaceae*		3.0923	8.8928
				*Clostridium IV*	0.3956	1.2496
				*Flavonifractor*	0.3921	1.3451
				*Oscillibacter*	0.7196	2.5839
				*Pseudoflavonifractor*	0.3489	1.3273
*Proteobacteria*					47.1815	27.1889
	*Betaproteobacteria*	*Neisseriales*	*Neisseriaceae*		1.5019	0.9755
				*Kingella*	1.3684	0.5617
	*Gammaproteobacteria*	*Pasteurellales*	*Pasteurellaceae*		13.3683	3.2420
				*Haemophilus*	11.4522	2.5269
				*Actinobacillus*	1.6611	0.5107
		*Pseudomonadales*	*Moraxellaceae*		30.2896	17.5549
				*Moraxella*	29.4876	17.0946
*Tenericutes*					5.1086	0.3500
	*Mollicutes*	*Mycoplasmatales*	*Mycoplasmataceae*		4.9747	0.0823
				*Mycoplasma*	4.9747	0.0823

## Discussion

Elucidation of the normal bacterial communities on body sites is critical for establishing differences associated with disease. In this study, the nasal microbiota composition of piglets was explored through direct sequencing of the 16S rRNA gene, which provided an overview of the nasal microbiota of healthy piglets, and the changes in this dynamic population associated to Glässer’s disease.

A few studies exist on the microbiota of the respiratory tract of pigs, although they mainly focused on tonsils or lungs [18, 40, 41], with only a recent study analyzing the nasal microbiota of healthy pigs [26]. Differential microbiota composition has been associated to several intestinal diseases [42], but this is the first report to study its relationship with Glässer’s disease, one of the main respiratory diseases of swine [43]. The nasal microbiome of healthy piglets contained a low diverse bacterial community in comparison with other body sites, such as gut [44]. In general, alpha diversity was found to be higher in control farms with no respiratory disease, C farms, when compared to GD farms. High microbial diversity is thought to be beneficial to the mucosal surfaces by reducing the opportunity for colonization by pathogens [45]. To our knowledge, this is the first report to relate microbiota diversity in the pig nares with Glässer’s disease, and is in agreement with many other studies where lower diversity correlated with disease [46–49].

Regarding the phylotypes found in pigs from C and GD farms, remarkable differences were found. A notably gain in *Proteobacteria* and loss in *Firmicutes* when the animals came from farms with Glässer’s disease was seen at phylum level. Interestingly, the *Pasteurallaceae* family was, at least in part, responsible for the higher amount of *Proteobacteria*, with *Haemophilus*, and particularly *H. parasuis*, being clearly increased. The fact that *H. parasuis* comprises strains with different degree of virulence makes necessary a deeper characterization of the virulence of the *specific* strains in the pigs to assess their role on Glässer’s disease outbreaks on each farm. As an example, the healthy status of farm VL could be explained by the lack of virulent *H. parasuis* strains in these animals (not detected by specific PCR [50], data not shown), since the global diversity of species in this farm was low and close to GD farms. Importantly, characterization of *H. parasuis* strains, in conjunction to the fact that they are statistically more abundant on diseased farms, could become useful as a non-invasive prognostic tool.

The prevalence of pathogens in the nares has been linked to increased risk of infection by other pathogens [51]. Relevant findings of the present study include the fact that the *Mycoplasmataceae* family was increased in GD farms, together with an increase in *Streptococcus* and *Haemophilus*. We hypothesized that the increased abundance of these pathogens could confer a higher risk to develop Glässer’s disease, but this should be experimentally demonstrated. On the other hand, *Moraxella* was shown to be a relevant component of the nasal microbiota, but it was found at higher abundance in diseased farms, declining the possibility of this genus to have a protective role in the nasal cavities, as it was hypothesized before [26]. However, we cannot rule out a role in protection with our results, since a deeper characterization of the *Moraxella* species and the precise strains present in the nasal cavities would be needed. In addition, the significant gain in *Proteobacteria* cannot be exclusively explained by these two genera mentioned (*Haemophilus* and *Moraxella*), and needs deeper research in order to understand the potential detrimental consequences, as many pathogens belong to this phylum.

More importantly, other relevant decreases were observed in some bacterial taxa from the nasal microbiota from GD farms, which might be predictive of beneficial commensals. This could be the case of both the *Ruminococcaceae* and the *Lachnospiraceae* families, which are also commensal members of the pig gut microbiota and have been associated to health in previous reports [52]. The same rational would apply to the order *Clostridiales*, which was reduced in GD farms and deserves also further attention. Moreover, the interaction between members of the gut microbiota and the respiratory system of the host have been reported [53, 54], conferring these data a more relevant interest as the gut microbiota can be manipulated with specific diet [55]. Additionally, the importance of pigs does not lay only in its relevance in food industry, but also in the physiological similarities to human, in terms of nutritional requirements and regarding the intestinal microbial ecosystem [20].

A phylogeny-based metric, UniFrac [37], was used to compare alpha diversity and assess the differences in overall bacterial community composition (beta diversity). This UniFrac-based principal coordinate analysis revealed clustering when the health status of the farm was evaluated. Clustering was observed when samples from the same country of origin were compared (Fig. 3a), although only around 10 % of the variability observed was explained, leaving a high amount of unexplained variation. This is typical for ecological studies [56] where it is not possible to control all environmental variables (e.g. external environmental factors, biological interactions, etc.). The clustering of samples from the same sow origin showed the highest percentage of variation. It is worth to mention that this group is composed of farms from a single company and with similar locations. We hypothesize that since the sows have the same origin, the predisposition to Glässer’s disease could not be associated to the genetic background, as it has been stated before for other diseases [31, 32], and the differences in microbiota would be due to other management factors. However, this hypothesis need further research, mainly due to the low number of the cohort study when they were subgrouped.

When clinical symptoms at about 8 weeks of age were considered for a GD farm (EJ farm), the beta diversity analysis evidenced individual differences in animals sharing the same environment, location, sow origin and other uncontrolled factors. Thus, the microbiota inhabitants of the nares of piglets may have an effect on the predisposition to develop Glässer’s disease, demonstrated by the fact that animals that remained healthy had similar microbiota composition to animals from C farms (Fig. 3a, red spheres with white dots).

Most of the differences observed in this study were detected by unweighted UniFrac metrics. It is reported that the abundance information in weighted UniFrac matrices do not consider the variation of the weights under random sampling, thus resulting in less power for detecting differences in communities [36, 37]. So the fact that almost no differences were detected in weighted UniFrac analysis can be related to those limitations, although it has to be considered that the qualitative information obtained through unweighted analysis can be emphasized by shallow OTUs.

Pigs are continuously rooting for food in farms, and this behavior could favor the transitory presence of microorganisms that will not finally colonize the nose. The analysis of the core microbiota was made with the aim of finding permanent inhabitants of the nasal cavities of pig (colonizers). The microbiota composition showed differences depending on the localization of the farms, as it was expected (Fig. 3b). However, those differences cannot be exclusively associated to country origin, as management practices, such as antibiotic treatments, were distinct too. Those OTUs in higher abundance in UK farms should be studied carefully, as these farms demonstrated higher diversity (Additional file 2), probably linked to their healthy status. In particular, farm KD was reported to eliminate respiratory problems after eliminating early antibiotic treatments [57], which may have an impact in increasing bacterial diversity.

The nasal microbiota composition of wild boars, which seem to be more resistant to Glässer’s disease, was explored for the first time and contrasted to the Spanish and British cores (Table 2). Interestingly, most of the OTUs found associated to health in Spanish farms were also found in similar abundance both in UK farms and wild boars, which are not exposed to massive use of antibiotics that can disturb the normal microbiota. These healthy animals showed to have more *Bacteroides,* pointing them as the main responsible of the differences observed. The exception of this was observed for *Haemophilus,* which was found in a high relative amount in wild boars, although being healthy animals. However, as stated before, the virulence of the specific strains needs to be investigated. This observation could imply the importance of the genetic background on predisposition to both acquisition of differential nasal microbiota and developing Glässer’s disease.

The information that provides this work, unraveling the differences among communities in the nasal mucosa, must be taken into consideration for future research related to Glässer’s disease control.

## Conclusion

Comparison of the nasal microbiota of piglets from farms experiencing Glässer’s disease and control farms revealed 8 OTUs (genus level) that were over-represented in the healthy animals, indicating a potential beneficial effect by these microorganisms. Besides, in a farm with disease, we detected differences in the nasal microbiota in animals that finally developed disease in comparison to those that remained healthy.

In conclusion, we were able to show through a confident non-invasive approach that the nasal cavity is colonized by a relatively diverse microbiota in healthy piglets. The nasal inhabitants showed to be in relation to clinical status of the farm of origin of the piglets, leading to different susceptibilities to invasive infection by *H. parasuis*. Several members have been identified as possible candidates to have a protective role against respiratory diseases and should be further studied. Although a 16S rRNA gene-based technique was used to reveal the microbial diversity in the nasal cavity, this approach offers only limited information on the physiological role of the microbial consortia. Fully metagenomic studies could give impressive information on the genes, metabolic pathways and resistances, among others characteristics related to the major roles that the microbiota plays in health and disease, giving more light on the every-day-less “forgotten organ” [58].

## Methods

### Sample collection

All procedures involving animals followed EU normative (Directive 2010/63/EU). Nasal swabs were taken from ten piglets at 3–4 weeks of age before weaning, from each farm. Two piglets per litter were randomly selected for sampling. Antibiotics used at 2–3 days after birth in the different farms are indicated on Table 4, together with other characteristics of the farms. Sampling was done from September 2014 to May 2015. All farms, except farm MC (which was negative), were positive for porcine reproductive and respiratory syndrome (PRRS) virus, but circulation of this virus was not diagnosed in the batch of piglets included in the study. Four farms where Glässer’s outbreaks were reported during 2013/2014 years, were sampled and considered as the diseased group (GD). In the control group, only farms with no respiratory conditions during the previous year of sampling and throughout the study, were included in the study (C) (Table 4). All the farms were located in the area of Catalonia (Spain), except for three farms in the control group that were from United Kingdom (UK). When possible, pigs were followed for 3 months after sampling, to gather information on clinical signs and healthy status. In addition, samples from three young wild boars (*Sus scrofa*) were used for comparison.

**Table 4:** Characteristics of the farms included in the study

Farm	Health status	Location	Sow farm size	Production system	Antibiotic treatment
MT	Glässer’s disease (GD)	Spain	3300	Multi-site	Pen + Strep
MC	Glässer’s disease (GD)	Spain	480	Farrow to finish	Ceft
RC	Glässer’s disease (GD)	Spain	1400	Multi-site	Ceft
EJ	Glässer’s disease (GD)	Spain	2000	Multi-site	Enro
VL	Control (C)	Spain	700	Farrow to finish	Tul + Ceft
PT	Control (C)	Spain	1000	Multi-site	NA
GM	Control (C)	Spain	1200	Multi-site	Amox
GW	Control (C)	UK	300	Farrow to finish	Amox
KD	Control (C)	UK	750	Multi-site	none
LG	Control (C)	UK	500	Farrow to finish	none

*Pen* Penicillin, *Strep* Streptomycin, *Ceft* Ceftiofur, *Enro* Enrofloxacin, *Tul* Tulathromycin, *Amox* Amoxicillin, *NA* Not available

Nasal swabs were taken from the nares of 10 animals and placed into a sterile tube. Swabs were transported to the laboratory on ice where they were resuspended in 500 μl of PBS and stored at −20 °C for further analysis.

### DNA extraction

Genomic DNA was extracted using the Nucleospin Blood (Machinery Nagel) kit. Briefly, frozen swabs were vortex vigorously and 200 μl were used for DNA extraction. Purified DNA was resuspended in a final volume of 50 μl of elution buffer (5 mM Tris, pH 8.5). The quality and quantity of genomic DNA was evaluated on a BioDrop DUO (BioDrop Ltd). All the extractions were done the same day the preparation of the library was done for sequencing purposes.

### 16S rRNA gene sequencing

Extracted DNA was sent to IBB facilities for Illumina pair-end 2X250 bp sequencing with MiSeq following the manufacturer instructions (MS-102-2003 MiSeq® Reagent Kit v2, 500 cycle).

The region targeted to perform the 16S amplification was the one spanning the V3 and V4 region of 16S rRNA gene selected from [59]. Interest-specific primers targeting this region were the ones recommended by Illumina with overhang adapters attached:

- 16S Forward Primer 5′ TCGTCGGCAGCGTCAGATGTGTATAAGAGACAGCCTACGGGNGGCWGCAG
- 16S Reverse Primer 5′ GTCTCGTGGGCTCGGAGATGTGTATAAGAGACAGGACTACHVGGGTATCTAATCC

Each of the primers contained a unique six-nucleotide barcode, so that the derived sequences can be sorted into the respective sample bioinformatically in downstream analysis. Cycling conditions included initial denaturation at 95 °C for 3 min followed by 25 cycles of 95 °C for 30 s, 55 °C for 30 s, and 72 °C for 30 s and a final extension at 72 °C for 5 min. The PCR product were purified and checked to verify its size on a Bioanalyzer DNA 1000 chip (Agilent).

Each sequence was assigned to its original sample according to its barcode. The barcode was removed from sequences before further processing, so that sequences that did not contain the barcode were eliminated.

### Taxonomic microbiota analysis

The QIIME (quantitative insights into microbial ecology [33]) software package (version 1.8) was used to process the reads following the protocols described by Kuczynski et al. [34] with some modifications. Firstly, reads were filtered in order to keep only high quality reads (Q > 25) as recommended in Bokulich et al. [60] for improving diversity estimates (using *split_libraries_fastq.py* script from QIIME). The high-quality-reads were paired-end joined by using fastq join [61, 62] using default values. OTUs were assigned at 97 % similarity using UCLUST algorithm [63] and the Greengenes database [64]. Sequences with dissimilarity higher than 3 % or lengths shorter than 350 bp were discarded from the analysis. A representative sequence from each of the OTUs, corresponding to the cluster seed, was subjected to taxonomic assignment with RDP database [65].

Samples were grouped according to health status (GD or C) initially, and further analyzed based on location and sow origin. Analysis was performed at different taxonomical levels (phylum, family and genus) separately. For each taxon g-test was performed between different groups and *p* values were FDR-corrected for multiple hypotheses testing (with *group_significance.py* QIIME script). Diversity indices (including Shannon-Wiener index, Faith’s PD Whole Tree and rarefaction curves) were calculated on rarefied 16S rRNA gene sequence data for all samples at 97 % similarity using QIIME (*alpha_diversity.py* script). Alpha diversity between groups was compared through non-parametric tests (Monte Carlo method) at maximum depth in rarefied samples (with 999 permutations) using *compare_alpha_diversity.py* QIIME script.

In addition, equal number of samples was subsampled to assess the significant differences between sample types using UniFrac weighted and unweigthed distances (*jackknifed_beta_divesity.py* QIIME workflow script) [37, 66]. The percentage of variation between grouped samples was measured by *R*^2^, using Adonis function of the vegan package in R software [35]. Estimation of *p*-values was obtained through Monte Carlo test with 999 random permutations of the data set (*compare_categories.py*, QIIME script). Samples were considered to be significantly different when the accompanying *P*-value was < 0.05.

## Abbreviations

C: control; GD: Glässer’s disease; OTU: operational taxonomic unit.

## Additional files

Additional file 1: Nasal microbiota of piglets farms with Glässer’s disease (GD) and control farms (C). The relative abundance (%) of OTUs found in nasal swabs of 3–4 weeks-old pigs is presented. Each graph represents the OTUs at different taxonomical levels: phylum (**a**), family (**b**), genus (**c**) for each sample. (TIFF 10882 kb) Additional file 2: Alpha diversity on rarefied samples analyzed by health status. Species richness of nasal samples from individual samples grouped by farm is shown. Dotted lines represent the standard deviation. (TIFF 25724 kb)
