# Supplementary figures and images for: Piglet nasal microbiota at weaning may influence the development of Glässer’s disease during the rearing period
Source: BMC Genomics. 2016 May 26;17:404. doi: 10.1186/s12864-016-2700-8 (PMC4881051; doi:10.1186/s12864-016-2700-8)

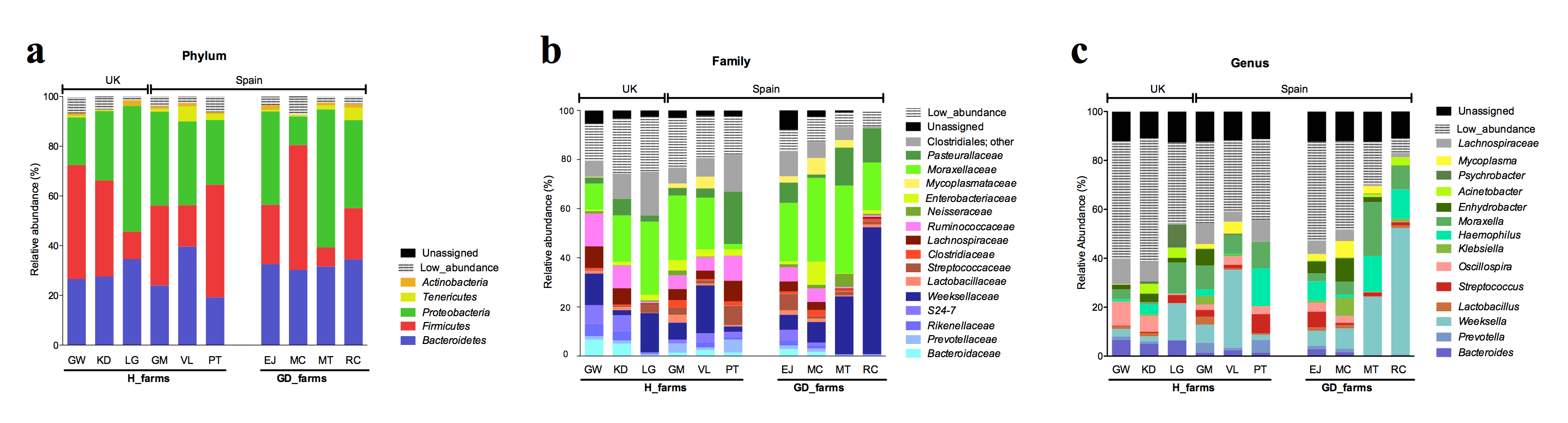

Supplement: Additional file 1: — Nasal microbiota of piglets farms with Glässer’s disease (GD) and control farms (C). The relative abundance (%) of OTUs found in nasal swabs of 3–4 weeks-old pigs is presented. Each graph represents the OTUs at different taxonomical levels: phylum (a), family (b), genus (c) for each sample. (TIFF 10882 kb) [file 12864_2016_2700_MOESM1_ESM.tiff]

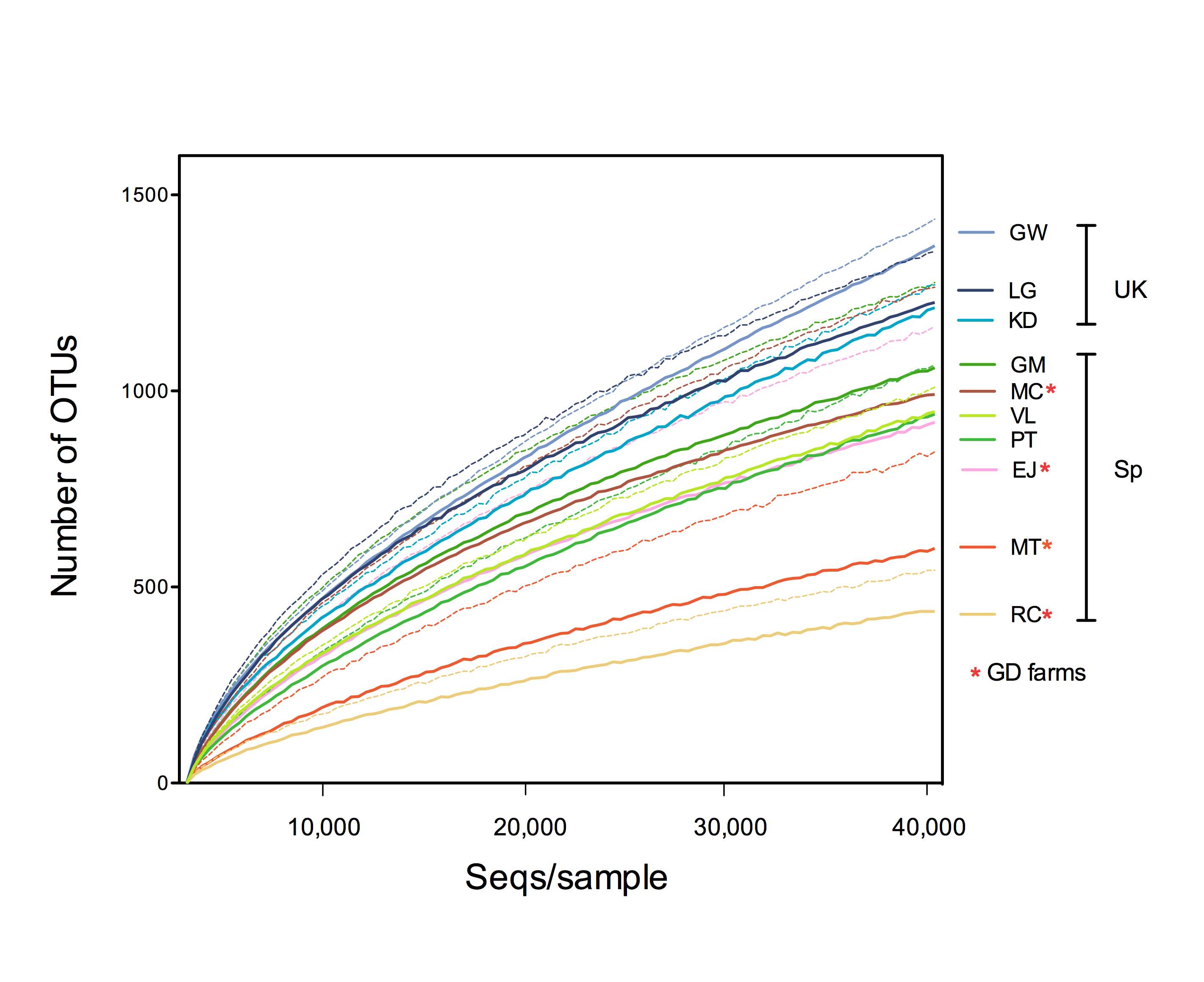

Supplement: Additional file 2: — Alpha diversity on rarefied samples analyzed by health status. Species richness of nasal samples from individual samples grouped by farm is shown. Dotted lines represent the standard deviation. (TIFF 25724 kb) [file 12864_2016_2700_MOESM2_ESM.tiff]
